# Cynaroside ameliorates methotrexate-induced enteritis in rats through inhibiting NLRP3 inflammasome activation

**DOI:** 10.3389/fimmu.2024.1405084

**Published:** 2024-05-21

**Authors:** Wuying Lang, Xin Wen, Shuangqi Zhang, Xuhua Liang, Lin Chen, Dezhu Zhang, Ruina Zhou, Ihsan Ali, Xuansheng Hu, Haihua Zhang, Min Cheng

**Affiliations:** ^1^ College of Biology Pharmacy and Food Engineering, Shangluo University, Shangluo, China; ^2^ Shaanxi Qinling Industrial Technology Research Institute of Special Biological Resources Co. Ltd., Shangluo, China; ^3^ Key Research Laboratory for Standardized Planting and Quality Improvement of Bulk Chinese Medicinal Materials in Shangluo, Shangluo University, Shangluo, China; ^4^ College of Pharmacy, Shaanxi University of Chinese Medicine, Xianyang, China; ^5^ Shaanxi Panlong Pharmaceutical Group Co. Ltd., Shangluo, China; ^6^ College of Veterinary Science Faculty of Animal Husbandry and Veterinary Science, The University of Agriculture Peshawar, Peshawar, Pakistan; ^7^ Hebei Key Laboratory of Specialty Animal Germplasm Resources Exploration and Innovation (Under Planning), College of Animal Science and Technology, Hebei Normal University of Science and Technology, Qinhuangdao, China

**Keywords:** cynaroside, intestinal inflammation, methotrexate, NLRP3, cleaved caspase 1, cleaved IL-1β

## Abstract

**Introduction:**

Cynaroside exhibits various biological properties, including anti-inflammatory, antiviral, antitumor, and cardioprotective effects. However, its involvement in methotrexate (MTX)-induced intestinal inflammation remains inadequately understood. Thus, we investigated the impact of cynaroside on MTX-induced intestinal inflammation and its potential mechanisms.

**Methods:**

To assess the protective potential of cynaroside against intestinal inflammation, Sprague-Dawley rats were subjected to a regimen of 7 mg/kg MTX for 3 days, followed by treatment with cynaroside at varying doses (10, 20, or 40 mg/kg). Histopathological evaluations were conducted alongside measurements of inflammatory mediators to elucidate the involvement of the NLRP3 inflammasome in alleviating intestinal inflammation.

**Results:**

Administration of 7 mg/kg MTX resulted in decreased daily food intake, increased weight loss, and elevated disease activity index in rats. Conversely, treatment with cynaroside at 20 or 40 mg/kg ameliorated the reductions in body weight and daily food intake and suppressed the MTX-induced elevation in the disease activity index. Notably, cynaroside administration at 20 or 40 mg/kg attenuated inflammatory cell infiltration, augmented goblet cell numbers and lowered serum levels of tumor necrosis factor-α, interleukin (IL)-1β, and IL-18, as well as the CD68-positive cell rate in the intestines of MTX-induced rats. Furthermore, cynaroside downregulated the expression levels of NLRP3, cleaved caspase 1, and cleaved IL-1β in MTX-induced rats.

**Discussion:**

Collectively, our findings indicated that cymaroside alleviates intestinal inflammatory injury by inhibiting the activation of NLRP3 inflammasome in MTX-induced rats.

## 1 Introduction

Methotrexate (MTX) is a widely used anticancer drug that significantly improves overall patient survival. However, MTX administration can induce intestinal mucosal inflammation, resulting in the impairment of intestinal barrier function, increased intestinal permeability, and other changes, thereby impacting treatment efficacy and posing the life-threatening risks to patients (1–3). The incidence of intestinal mucositis associated with standard-dose MTX chemotherapy is approximately 40%, with high-dose MTX chemotherapy elevating this rate to approximately 100% (4, 5). Notably, regular damage patterns and reduction in the length of small intestinal microvilli have been observed during MTX treatment, accompanied by alterations in the physical structure of the brush border membrane (6). Upon MTX exposure, intestinal cells release pro-inflammatory cytokines such as tumor necrosis factor (TNF)-α and interleukin (IL)-6 (7), which, in turn, can induce the activation of the NLR family pyrin domain containing 3 (NLRP3) inflammasome and subsequent release of pro-inflammatory cytokines such as IL-18 and IL-1β (8). NLRP3 is highly expressed during intestinal inflammation and tissues damaged (9). We previously reported associations between intestinal inflammation and the NLRP3 inflammasome (10), suggesting that the development of NLRP3-targeted therapeutics may hold promise in mitigating enteritis during MTX chemotherapy.

Cynaroside, also known as luteolin-7-O-glucoside, is a flavonoid compound widely distributed in medicinal plants of the *Lonicera* family. Cynaroside exhibits diverse biological activities, including anti-inflammatory, antiviral, antitumor, and cardio- and neuroprotective properties (11). It reportedly mitigates mucositis by modulating downstream signaling of the NLRP3 inflammasome or related inflammatory factors. In addition, cynaroside suppresses the expression of IL-22 and IL-6 through the activation of the JAK/STAT3 pathway and inhibition of pro-inflammatory gene transcription (12). Li et al. reported that cynaroside treatment attenuated ischemia and reperfusion-induced neuroinflammation by reducing the levels of IL-1β and TNF-α and inhibiting nuclear factor-kappa B (NF-κB) pathway activation in brain tissues of rats subjected to middle cerebral artery occlusion (11). Moreover, cynaroside alleviated osteoarthritis by inhibiting IL-1β-induced phosphorylation of mitogen-activated protein kinases and nuclear translocation of the NF-κB p65 subunit (13). However, most investigations on cynaroside have centered on its potential antitumor effects (14–16), with limited evidence available regarding its protective effects against intestinal inflammation.

Therefore, in the current study, we aimed to investigate the anti-inflammatory effects and the potential mechanism of cynaroside in MTX-induced rats’ intestinal inflammatory injury.

## 2 Materials and methods

### 2.1 Reagents and antibodies

Cynaroside (>98% purity) was procured from MedChemExpress (Shanghai, China), and MTX (>98% purity) was obtained from Beijing Solarbio Science & Technology Co., Ltd. (Beijing, China). ELISA kits for TNF-α, IL-1β, and IL-18, DAPI, Cy3-labeled Goat Anti-Rabbit IgG (H+L), and antibodies against Cy3-labeled Goat Anti-Mouse IgG (H+L) (B100801) were sourced from Jiangsu Meimian Industry Co., Ltd. (Jiangsu, China). Antibodies against glyceraldehyde 3-phosphate dehydrogenase (GAPDH) and NLRP3 were acquired from Abcam (Cambridge, UK), while antibodies against cleaved caspase1 and cleaved IL-1β were purchased from Cell Signaling Technology (Boston, USA). The CD68 antibody was obtained from Santa Cruz Biotechnology (Shanghai, China), and the NLRP3 antibody was sourced from Affinity (Jiangsu, China). Horseradish peroxidase (HRP)-Goat Anti-Rabbit and Goat Anti-Mouse antibodies were purchased from KPL (Gaithersburg, MD, USA).

### 2.2 Animal model establishment and cynaroside treatment

Thirty Sprague-Dawley adult male rats with an average weight of 211.38 ± 3.25 g, were adaptively housed in an experimental animal facility for one week under controlled conditions (25 ± 1 °C, 40–80% humidity, with ad libitum access to pathogen-free food and water) under a 12:12 light/dark cycle. Rats were randomly allocated to five groups (n = 6/group): Control, MTX, MTX + Cynaroside (10 mg/kg), MTX + Cynaroside (20 mg/kg), and MTX + Cynaroside (40 mg/kg). Cynaroside concentrations were determined based on previous studies (11). For MTX administration, rats were intraperitoneally administered 7 mg/kg MTX for 3 consecutive days. Control group rats received an equivalent volume of normal saline. Regarding cynaroside treatment, rats were administered 10, 20, or 40 mg/kg cynaroside by gavage for 7 days during MTX treatment. Twenty-four hours after the final administration, rats were anesthetized by administering 2% sodium pentobarbital intraperitoneally, followed by the collection of blood samples and small intestinal tissue samples. All animal experimental procedures were conducted in accordance with the guidelines approved by the Ethics Committee of Shangluo University. The study was conducted in accordance with the local legislation and institutional requirements.

### 2.3 Disease activity index assays

During the experimental period, three observers monitored the body weight, fecal occult blood, and stool characteristics to determine the DAI, as described previously (10).

### 2.4 Biochemical assays

Blood samples were centrifuged at 1,500 × g for 15 min at 4 °C. Serum TNF-α, IL-1β, and IL-18 levels were quantified using commercial ELISA kits following the manufacturer’s protocol.

### 2.5 Histopathological evaluation

In brief, fixed rat small intestinal tissues were embedded in paraffin and sectioned. These sections were stained with hematoxylin and eosin (H&E) or periodic acid-Schiff (PAS) and were subsequently examined under a microscope (Olympus Corporation, Tokyo, Japan) at 200× magnification. Inflammation severity was graded on a scale from 0 to 4: 0, none; 1, rare; 2, common; 3, frequent; and 4, severe (10). PAS-positive cells were quantified using Image Pro Plus v6.0 (Media Cybernetics, Inc., Rockville, MD, USA).

### 2.6 Immunofluorescence analysis

A subset of the prepared sections were subjected to immunofluorescence analysis. These Section were incubated with primary antibodies against CD68 (1:200) or NLRP3 (1:200) at 4 °C overnight after blocking with 5% bovine serum albumin for 30 min at room temperature. Section were then incubated with the secondary antibody, either Cy3-labeled Goat Anti-Mouse IgG (H+L) (1:200) or Cy3-labeled Goat Anti-Rabbit IgG (H+L) (1:200) in the dark for 50 min at room temperature; nuclei were stained with DAPI for 15 min in the dark. The sections were observed under a fluorescence microscope (200x, Olympus Corporation, Tokyo, Japan) and quantified using Image Pro-Plus 6.0 (Media Cybernetics, Silver Spring, USA).

### 2.7 Western blotting

Proteins were extracted from the small intestinal tissue, as described previously (10). Proteins were separated using performing sodium dodecyl sulfate-polyacrylamide gel electrophoresis and transferred to polyvinylidene difluoride membranes. After blocking with 5% nonfat milk for 1 h at room temperature, membranes were incubated with primary antibodies against GAPDH (1:6,000), cleaved caspase1 (1:1,000), cleaved IL-1β (1:1,000), and NLRP3 (1:1,000) overnight at 4 °C. Next, the membrane was washed four times with TBST, and incubated with Goat Anti-Mouse IgG (H+L) (1:50,000) or Goat Anti-Rabbit IgG (H+L) (1:50,000) for 30 min at room temperature. Protein bands were visualized using an enhanced chemiluminescence (ECL) kit (Jiangsu Meimian Industry) and quantified with ImageJ software (National Institutes of Health, USA). GAPDH was used as an internal reference.

### 2.8 Statistical analysis

Data are presented as the mean ± standard deviation (SD). Statistical analyses were conducted using SPSS software, version 26.0 (IBM Corp., Armonk, NY, USA). Significant differences among groups were determined using one-way ANOVA. A *P*-value of < 0.05 was deemed statically significant.

## 3 Results

### 3.1 Cynaroside alleviates the decline in growth performance in MTX-induced rats

**Figure 1:**
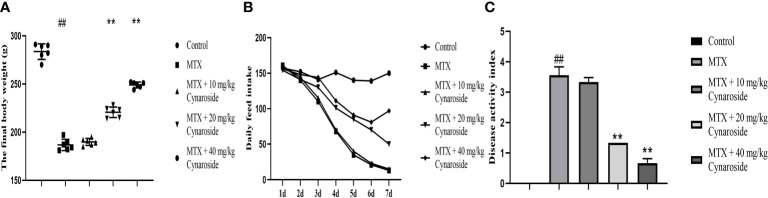
Cynaroside alleviates the decline in growth performance in MTX-induced rats. **(A)** Final body weight in the different treatment groups. **(B)** Daily food intake in the different treatment groups. **(C)** DAI in the different treatment groups. Data are expressed as the mean ± SD. n = 6. ##*P* < 0.01, compared with the control group. **P < 0.01, compared with the MTX group.

The final body weight of rats in the MTX group was significantly lower than that of rats in the control group. However, treatment with cynaroside at 20 or 40 mg/kg significantly restored the loss of body weight (*p* < 0.01; **Figure 1A**). Daily food intake decreased gradually in the MTX group, whereas cynaroside (40 mg/kg) increased daily food intake in MTX-induced rats (**Figure 1B**). Moreover, the DAI of rats supplemented with 20 or 40 mg/kg cynaroside was significantly lower than that of MTX-induced rats (*p* < 0.01, **Figure 1C**).

### 3.2 Cynaroside attenuates intestinal histopathological changes in MTX-induced rats

**Figure 2:**
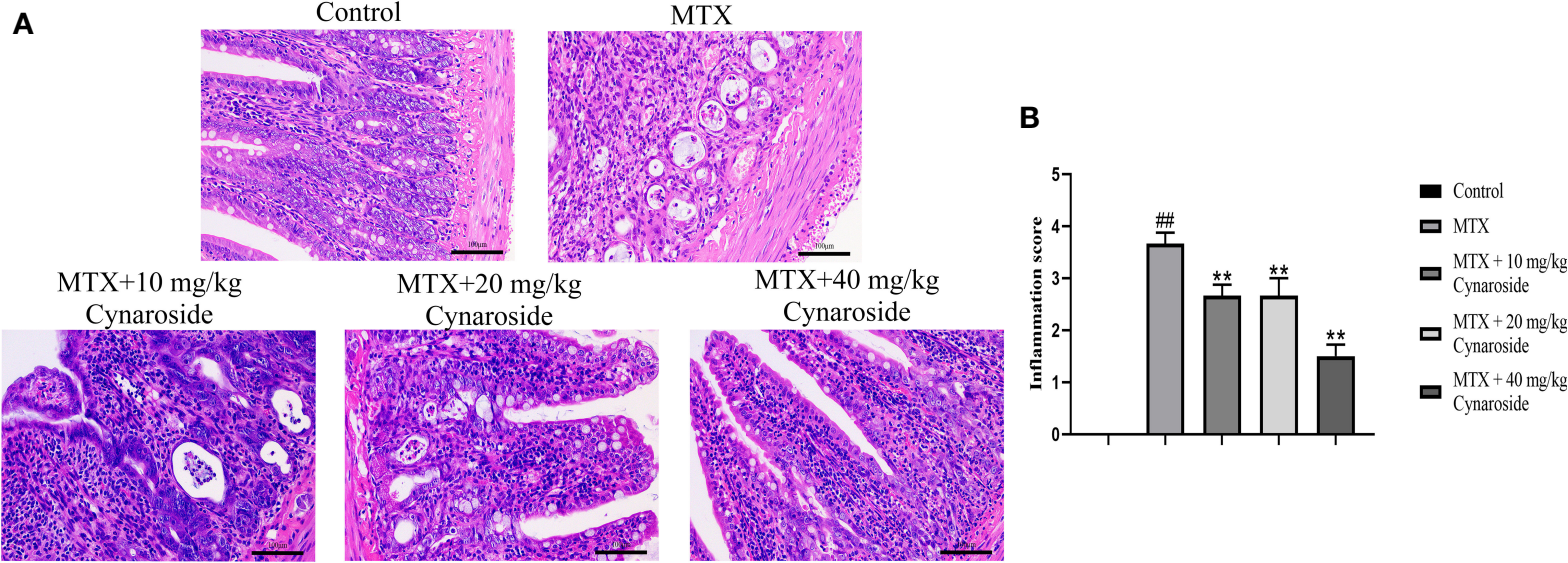
Effect of cynaroside on intestinal pathological alterations in MTX-induced rats. **(A)** Representative small intestine tissue of histopathological H&E staining (200x). **(B)** Statistical analysis of the inflammation score. Data are expressed as the mean ± SD. n = 6. ##*P* < 0.01, compared with the control group. **P < 0.01, compared with the MTX group. Scale bars represent 100 μm.

H&E staining was performed to examine histological alterations in the small intestine (**Figure 2A**). Rats in the MTX group exhibited typical pathological changes, including inflammatory cell infiltration, mucosal layer destruction, gland expansion, and intestinal villus structure disorder, compared with those in the control group. Treatment with cynaroside significantly ameliorated the MTX-induced morphological alterations; rats administered 40 mg/kg cynaroside showed intact intestinal tissues without inflammatory features. Additionally, cynaroside reduced the intestinal inflammation score (**Figure 2B**).

### 3.3 Cynaroside enhances the number of intestinal goblet cells in MTX-induced rats

**Figure 3:**
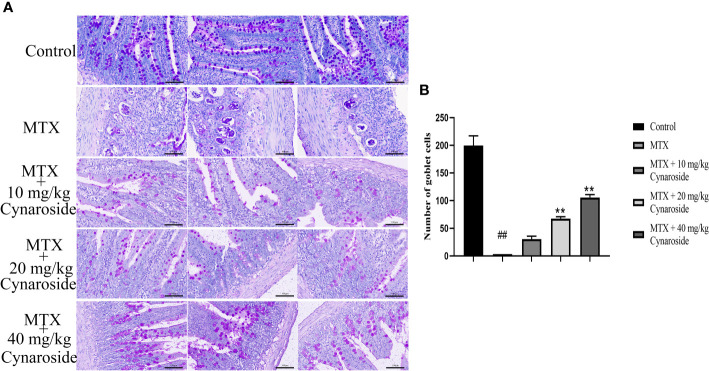
Effect of cynaroside on the small intestinal goblet cells in MTX-induced rats. **(A)** Goblet cells were assessed by PAS staining (200x). **(B)** Statistical analysis of the number of goblet cells. Data are expressed as the mean ± SD. n = 6. ##*P* < 0.01, compared with the control group. **P < 0.01, compared with the MTX group. Scale bars represent 100 μm.

PAS staining was used to evaluate the effect of cynaroside on the small intestinal goblet cells (**Figure 3A**). The number of goblet cells in the small intestine significantly decreased in the MTX-induced group compared with that in the control group (*p* < 0.01, **Figure 3B**). However, treatment with cynaroside, particularly at a dose of 20 or 40 mg/kg, increased the number of goblet cells in MTX-induced rats (*p* < 0.01, **Figure 3B**).

### 3.4 Cynaroside suppresses the inflammatory response in MTX-induced rats

**Figure 4:**
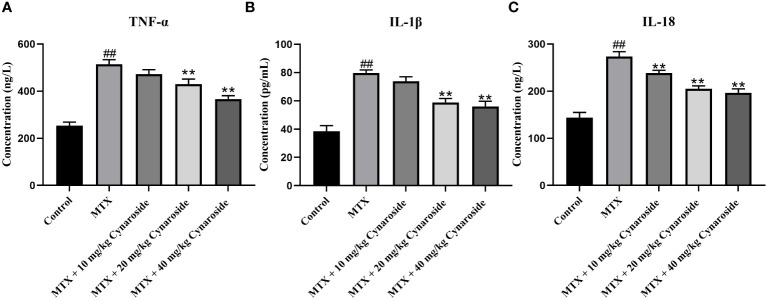
Effect of cynaroside on serum pro-inflammatory factors levels in MTX-induced rats. Serum levels of **(A)** TNF-α, **(B)** IL-1β, and **(C)** IL-18 as assessed using by ELISA. Data are expressed as the mean ± SD. n = 6. ##*P* < 0.01, compared with the control group. **P < 0.01, compared with the MTX group.

The serum levels of representative pro-inflammatory cytokines TNF-α, IL-1β, and IL-18 were significantly upregulated in the MTX-induced group compared with those in the control group (all p < 0.01, **Figures 4A-C**). However, these levels were significantly reduced in the cynaroside-treated groups (20 or 40 mg/kg) compared with those in the MTX-induced group (all p < 0.01).

### 3.5 Cynaroside reduces the amount of CD68 in MTX-induced rats

**Figure 5:**
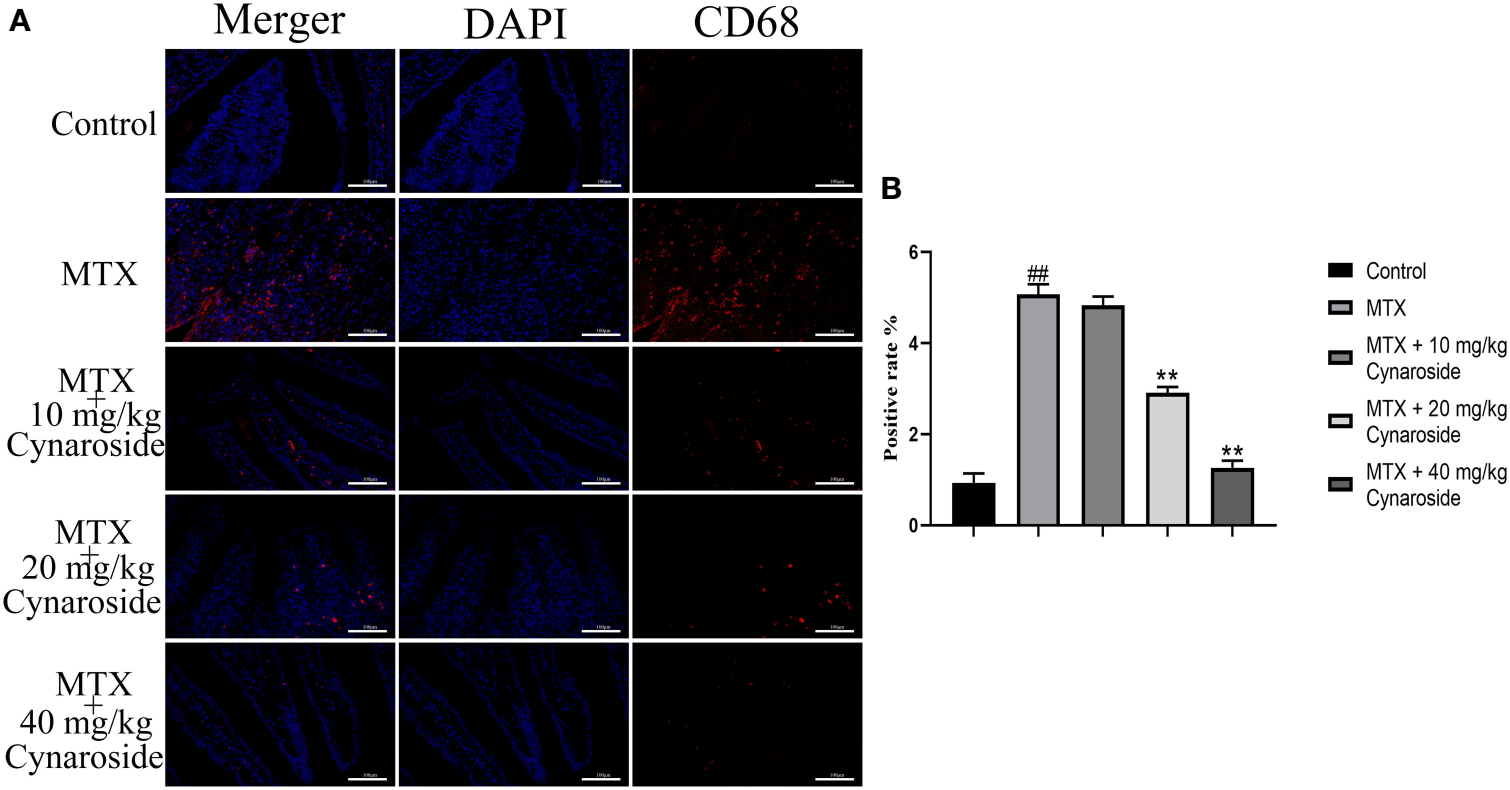
CD68-positive cell rate in the small intestine. **(A)** Immunofluorescence analysis of CD68-positive cells in the different treatment groups (200x). **(B)** CD68-positive rate in the different treatment groups. Data are expressed as the mean ± SD. n = 6. ##*P* < 0.01, compared with the control group. **P < 0.01, compared with the MTX group. Scale bars represent 100 μm.

Next, we examined CD68, a marker of macrophage/monocyte infiltration using immunofluorescence staining. The number of CD68-positive cells was significantly higher in the MTX-induced group than that in the control group. Conversely, treatment with 20 or 40 mg/kg cynaroside significantly reduced the number of CD68-positive cells (**Figures 5A, B**).

### 3.6 Cynaroside inhibits NLRP3 inflammasome activation in MTX-induced rats

**Figure 6:**
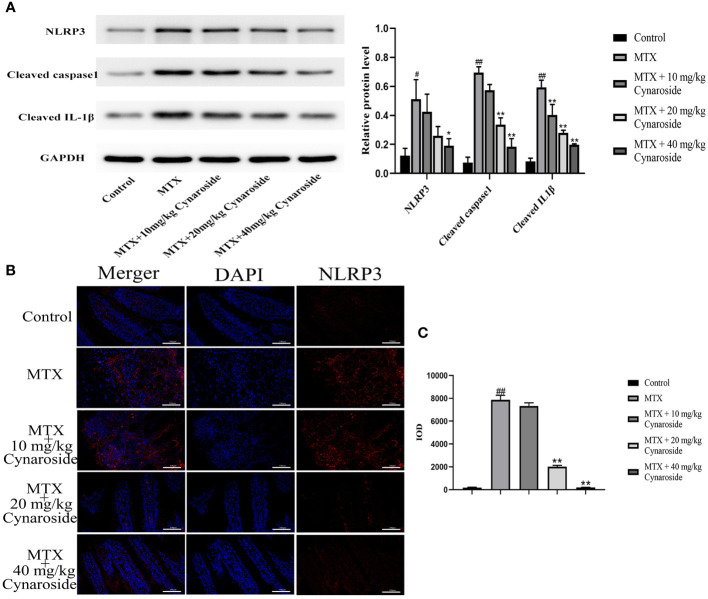
Effect of cynaroside on the NLRP3 inflammasome in MTX-induced rats. **(A)** the expression of NLRP3, cleaved caspase 1, cleaved IL-1β as measured by western blot. GAPDH was used as a control. **(B)** NLRP3 immunofluorescence (200x). **(C)** Immunofluorescence cumulative optical density (IOD) of NLRP3. Data are expressed as the mean ± SE. n = 3. #*P* < 0.05, ##*P* < 0.01 compared with the control group. *P < 0.05, **P < 0.01, compared with the MTX group. Scale bars represent 100 μm.

The activation of the NLRP3 inflammasome is pivotal in intestinal inflammation. Hence, we examined alterations in NLRP3 inflammasome-associated factors using western blotting and immunofluorescence analysis. The results showed that the expression of NLRP3, cleaved caspase 1, and cleaved IL-1β significantly increased in MTX-induced rats, suggesting that MTX treatment activates NLRP3 inflammasome (**Figures 6A, B**). Treatment with 20 or 40 mg/kg cynaroside restored the expression of these molecules (**Figures 6A-C**). Collectively, these findings suggest that cynaroside could effectively inhibit NLRP3 inflammasome activation in MTX-induced rats.

## 4 Discussion

MTX is a folate antagonist that inhibits cell growth and reproduction by inhibiting dihydrofolate reductase (17, 18). It is widely used to treat acute leukemia, various solid tumors, and autoimmune diseases, and is the gold standard drug for rheumatoid arthritis (19, 20). However, MTX administration can lead to a range of complications, including gastrointestinal injury and bone marrow suppression (21–23). MTX-induced intestinal mucositis is a common gastrointestinal complication associated with symptoms such as diarrhea, infections, and bloody stools (24, 25). However, effective strategies to mitigate or alleviate severe gastrointestinal complications induced by MTX, particularly high-dose MTX, remain to be established. In the current study, we established an intestinal inflammatory model using non-tumor-bearing rats. We observed that MTX administration decreased daily feed intake and final body weight and increased the DAI score in rats. Intestinal pathological examination revealed various inflammatory phenomena, including inflammatory cells infiltration, mucosal layer destruction, gland expansion, disordered intestinal villi structure, and a reduction in goblet cell numbers in the small intestine of MTX-induced rats. Our findings are consistent with those reported previously, indicating that high-dose MTX induces intestinal mucositis (24, 25).

Luteolin is a common flavonoid found in Chinese medicinal herbs and is present in its glycosylated form. Cynaroside is a primary active component of luteolin (26). which has been shown to exert anti-inflammatory effects (27). We observed that cynaroside attenuated the MTX-induced reductions in daily feed intake, weight, and goblet cell numbers and improved the DAI score in rats. Furthermore, cynaroside inhibited the MTX-induced pathological damage to the intestinal mucosa. MTX substantially increased the serum levels of TNF-α, IL-1β, and IL-18, whereas cynaroside suppressed the inflammatory response, demonstrating its anti-inflammatory effect in MTX-induced intestinal mucositis. Our findings align with those of a previous study, indicating that cynaroside significantly inhibits ischemia and reperfusion-induced neuroinflammation. This was evidenced by reductions in IL-1β, TNF-α, inducible nitric oxide synthase (iNOS), and cyclooxygenase 2 (COX-2) levels in brain tissues of rats with MCAO, along with suppression of the NF-κB signaling activation (11). In a study on *Staphylococcus aureus*-induced endometritis, treatment with cynaroside was found to downregulate the expression of pro-inflammatory factors TNF-α, IL-1β, and IL-6, and upregulate the expression of the anti-inflammatory cytokine IL-10, while suppressing the TLR2 and NF-κB signaling pathways (28). Cynaroside suppressed the expression levels of TNF-α, and IL-6, as well as iNOS, and COX-2, in lipopolysaccharide-induced RAW264.7 cells while inhibiting NF-κB activation by suppressing the phosphorylation and degradation of IκB-α (29). Moreover, cynaroside decreased the expression of IL-1β, IL-6 and TNF-α,and inhibited HMGB1 in cecal ligation and puncture-induced liver inflammation (30). Additionally, CD68+ macrophages, which are significant sources of inflammatory cytokines in inflammatory bowel disease (IBD) (31). Herein, we showed that cynaroside could reduce the MTX-induced increase in CD68 expression in the small intestines of rats. Based on previous studies and our results, cynaroside may exert potent anti-inflammatory effects.

The NLRP3 inflammasome plays a significant role in the immune system and IBD being highly expressed during intestinal inflammation (32–34). Its activation promotes the release of pro-inflammatory cytokines IL-1β and IL-18 (9). The role of the NLRP3 inflammasome corpuscles in IBD pathogenesis and progression has been extensively studied, with efforts focused on the developing novel targeted therapies to inhibit it in IBD (35, 36). Blockers targeting specific components of the NLRP3 pathway are under consideration for clinical application in the treatment of various inflammatory diseases (37). Notably, inhibiting NLRP3 inflammatory bodies has been shown to prevent the onset of enteritis (31). Our findings consistently demonstrated that cynaroside can inhibit the MTX-induced NLRP3 inflammasome activation, as evidenced by the reductions in NLRP3, cleaved caspase-1, and mature IL-1β, and IL-18 expression.

In conclusion, our findings suggest that cynaroside can alleviate intestinal inflammation by reducing the serum pro-inflammatory factors in serum, improving intestinal pathological injury, and inhibiting the expression of NLRP3 inflammasome-related proteins. These findings highlight the potential of cynaroside as a therapeutic agent for treating intestinal inflammation. Additionally, future studies could comprehensively investigate the action mechanisms underlying anti-inflammatory effects of cynaroside and potentially help identify novel therapeutic targets for the treatment of intestinal inflammation.

## Data availability statement

The datasets presented in this study can be found in online repositories. The names of the repository/repositories and accession number(s) can be found in the article/**Supplementary Material**.

## Ethics statement

The animal study was approved by Ethics Committee of Shangluo University. The study was conducted in accordance with the local legislation and institutional requirements.

## Author contributions

WL: Conceptualization, Data curation, Formal analysis, Writing – original draft, Writing – review & editing. XW: Data curation, Formal analysis, Writing – review & editing. SZ: Data curation, Formal analysis, Writing – review & editing. XL: Conceptualization, Data curation, Writing – review & editing. LC: Data curation, Formal analysis, Project administration, Writing – review & editing. DZ: Data curation, Formal analysis, Project administration, Writing – review & editing. RZ: Data curation, Formal analysis, Project administration, Writing – review & editing. IA: Data curation, Formal analysis, Software, Writing – review & editing. XH: Data curation, Formal analysis, Software, Writing – review & editing. HZ: Data curation, Formal analysis, Software, Writing – review & editing. MC: Conceptualization, Writing – review & editing.
