# Supplementary material for: Cynaroside ameliorates methotrexate-induced enteritis in rats through inhibiting NLRP3 inflammasome activation
Source: Front Immunol. 2024 May 21;15:1405084. doi: 10.3389/fimmu.2024.1405084 (PMC11148340; doi:10.3389/fimmu.2024.1405084)
Supplement: Supplementary file 1 [file DataSheet_1.docx]

**Supplementary material**

The datasets [original data, images, and analyzed] for this study can be found in the [Raw data] [https://www.jianguoyun.com/p/DbxQOsQQ_Ja6DBjXv7oFIAA, <https://www.jianguoyun.com/p/Dei0nUgQ_Ja6DBjbv7oFIAA,> <https://www.jianguoyun.com/p/DZtvD0cQ_Ja6DBj1h7wFIAA,> https://www.jianguoyun.com/p/De7hbtsQ_Ja6DBiZiLwFIAA].
